# Massive envenomations by bees in French Guiana: A ten-year retrospective study (2014–2023)

**DOI:** 10.1371/journal.pntd.0014824

**Published:** 2026-10-23

**Authors:** Rémy Blaud, Rémi Mutricy, Florian Negrello, Marie Eeckhout, Loïc Epelboin, Maylis Douine, Hatem Kallel, Alexis Fremery

**Affiliations:** 1 Emergency Department, Centre Hospitalier Universitaire de Guyane, Cayenne, French Guiana, France; 2 University Department of General Practice, Université des Antilles-Guyane, Pointe-à-Pitre, Guadeloupe, France; 3 Emergency Department, Centre Hospitalier Universitaire de Martinique, Fort-de-France, Martinique, France; 4 Infectious and Tropical Diseases Unit, Centre Hospitalier Universitaire de Guyane, Cayenne, French Guiana, France; 5 Centre d’Investigation Clinique Guyane – INSERM 1424, UMR UA 17 Santé des Populations en Amazonie, Centre Hospitalier Universitaire de Guyane, Cayenne, French Guiana, France; 6 Intensive Care Unit, Centre Hospitalier Universitaire de Guyane, Cayenne, French Guiana, France; HIA Begin: Hopital d’Instruction des Armees Begin, FRANCE

## Abstract

**Background:**

Massive bee stings can cause systemic manifestations and life-threatening complications. In French Guiana, data on such envenomations are scarce. This study aimed to describe the epidemiology, clinical characteristics, and management of patients presenting to the emergency department.

**Methods:**

We retrospectively reviewed medical records of all patients admitted for bee stings over the period 2014–2023 at Cayenne Hospital. A composite variable of systemic involvement was created (neurological, respiratory, hemodynamic and digestive). All analyses were descriptive. An exploratory two-graph receiver operating characteristic (TG-ROC) analysis was used to describe the sensitivity and specificity of sting-count cut-offs for four binary indicators of systemic involvement.

**Results:**

Among 143 patients included, 40% (n = 57) presented at least one systemic manifestation. The median number of stings was 50 [20–150]. In the exploratory TG-ROC analysis, the cut-offs maximizing the Youden index were approximately 72 stings for digestive and hemodynamic manifestations and 99 stings for neurological and respiratory manifestations, with modest sensitivity (0.50–0.76) and specificity (0.68–0.76). Overall mortality was low (0.7%, n = 1/143).

**Conclusions:**

This study provides the first detailed epidemiological and clinical description of massive bee envenomations in French Guiana. Exploratory analyses suggested that systemic manifestations may occur at sting counts below the conventional 200-sting threshold. These findings should not be interpreted as a validated clinical cutoff and require confirmation in prospective multicenter studies.

## Author summary

Most bee stings cause only local pain and swelling. However, being stung many times can lead to serious illness because of the large amount of venom injected. Few studies have described these multiple-sting attacks in French Guiana. We reviewed the medical records of 143 patients treated at Cayenne Hospital after multiple honey bee stings between 2014 and 2023. Most patients were men, and the median number of stings was 50. Forty percent of patients developed at least one systemic manifestation, such as breathing problems, low blood pressure, severe digestive symptoms, or neurological symptoms. Our results suggest that such symptoms may occur after fewer than the 200 stings traditionally considered to indicate severe envenomation. However, this observation needs to be confirmed, as our study was retrospective and based on patients treated at a single hospital, and the number of stings was estimated from medical records. These findings suggest that people who have been stung many times should be assessed carefully, even when the number of stings is below 200. Further studies are needed to confirm these results.

## Introduction

The Africanized honey bee, also known as the “killer bee,” has been responsible for numerous casualties across the Americas since its introduction. In Brazil, hundreds of attacks have been recorded, and Venezuela has reported over 400 deaths in 15 years [1,2]. This subspecies is a hybrid resulting from the crossbreeding of European honey bees (*Apis mellifera iberica* or *Apis mellifera mellifera*, introduced by the Portuguese in the 15th century) and the African subspecies *Apis mellifera scutellata* (introduced in the 20th century) [3]. The resulting hybrid has shown high aggressiveness, frequent swarming behavior, and low defensiveness thresholds [4]. These traits have allowed it to spread across nearly the entire American continent in less than 40 years [5]. Today, all honey bee populations of the *Apis mellifera* genus exhibit varying degrees of hybridization with *scutellata in south America and especially in French Guiana* [5].

Compared to their European counterparts, Africanized bees react more quickly to pheromonal signals and can recruit up to 50% of the hive for defense, versus only 10% for European bees [6]. Their colonies sting 4–10 times more frequently and may pursue intruders with 10–30 times more bees [7,8]. In the literature, an attack involving more than 200 stings is considered severe, and it may become potentially fatal above 500 stings in adults and 30 in children [3]. Bee stings can cause three types of reactions. The first is a local inflammatory reaction characterized by an immediate painful, warm, erythematous papule at the site of the sting. The second is an immediate hypersensitivity allergic reaction, which may include generalized symptoms such as urticaria, abdominal pain, nausea, vomiting, diarrhea, hypotension, dyspnea, hypoxia, or even anaphylactic shock. The third is a delayed systemic toxic reaction (48–96 hours) due to massive envenomation and high venom load [3]. These mechanisms may overlap, particularly after multiple stings, in which allergic and venom-mediated systemic manifestations may coexist. This toxic response can also lead to rhabdomyolysis, acute renal failure, and multiorgan dysfunction [6,9]. Bee venom contains various allergenic and toxic components, the main allergen being phospholipase A2, and dose-dependent toxins such as melittin and hyaluronidase [5].

Bees tend to thrive in open vegetated areas. In French Guiana, a French territory located in the Amazon region, 96% of the territory is covered by dense tropical rainforest. However, the coastal strip consists mainly of open savannas where most of the population resides. The increasing population density is driving urban expansion into previously forested areas, which raises the frequency of human-wildlife interactions—especially with hymenopterans, which are a leading cause of envenomation [10].

The aim of this study was to establish the epidemiology of mass stinging events caused by bees presumed to be Africanized honey bees in French Guiana. Secondary objectives were to describe the systemic manifestations of these envenomations, to explore descriptively their distribution according to the number of stings, and to describe the clinical management of the victims.

## Materials and methods

### Ethical statement

The study was conducted in accordance with French data protection regulations. A declaration of compliance with the MR-004 reference methodology of the French Data Protection Authority (CNIL) No. 2231742 was filed. A collective information notice was provided to patients. The study was registered in the hospital’s internal data processing register and on the Health Data Hub platform. All data were anonymized and collected retrospectively using a standardized form, in accordance with CNIL guidelines, ensuring the absence of personally identifiable information.

### Study design

This was a retrospective, descriptive, monocentric study conducted at the Emergency Department of the Cayenne General Hospital (French Guiana) from January 1, 2014, to December 31, 2023.

### Study population

The study population included all patients admitted to the Emergency Department following an attack by at least five honey bee stings, identified as bees either by the patient or by the medical team. The estimated number of stings was extracted from the emergency department medical records. As this was a retrospective study, the estimate corresponded to the number documented on the patient’s report. The method used to estimate the sting count was not systematically recorded. The threshold of five stings was not intended to exclude allergic reactions, which may occur irrespective of the number of stings, but rather to focus the study on multiple-sting envenomation. Toxic envenomation is not expected after only one or a few stings, whereas multiple stings result in a substantially greater venom load and represent the clinical situation of interest in this study. Patients with allergic manifestations were therefore not excluded if they fulfilled the inclusion criteria. Cases involving unidentified hymenopterans or other insects from the same order (e.g., wasps, hornets, ants) were excluded.

### Data collection

Data were extracted from three medical software systems used at Cayenne General Hospital:

- DMU (Données Médicales des Urgences / *Emergency Medical Data*): the hospital’s internal emergency medical record system, used to retrieve consultation reasons and initial diagnoses.
- SRI (Service des Résultats Intranet / *Intranet Results Service*): a secure online platform for accessing laboratory and pathology results.
- CORA Document: the hospital’s electronic system for storing hospitalization discharge summaries.

A keyword search was performed in the DMU software for the period between January 1, 2014, and December 31, 2023, using a broad set of terms to ensure sensitivity: “bee,” “insect,” “hymenoptera,” “allergy,” “anaphylaxis,” and “sting.” Each medical record was individually reviewed to confirm the species involved and the estimated number of stings. Incomplete cases (e.g., patients who left without being seen or with missing data) were excluded.

The following data were collected: Demographics (age and sex); Contextual details (date and circumstances of the attack); Clinical and biological consequences; Prehospital and hospital management; Patient outcomes.

Bees were identified as honey bees based on patient or clinician report. No entomological or genetic confirmation was available. Given the known presence and distribution of Africanized honey bees in the region, the attacks were considered presumed Africanized honey bee stings; however, misclassification with other honey bee populations or bee species cannot be excluded.

### Statistical analysis

Data analysis was conducted using Microsoft Excel (v16.100.3) and R Studio (v2023.06.0 + 421). Categorical variables were described as frequencies and percentages. Continuous variables were expressed as means ± standard deviation (SD) when normally distributed, or as medians [Q1; Q3] when not normally distributed.

Systemic manifestations were defined using the following operational criteria:

- Neurological manifestations: Glasgow Coma Scale <15 or seizure.
- Respiratory manifestations: oxygen saturation <95% and/or oxygen therapy.
- Hemodynamic manifestations: mean arterial pressure <65 mmHg and/or intravenous fluid resuscitation.
- Gastrointestinal manifestations: severe gastrointestinal involvement requiring intravenous fluids or hospitalization.

Systemic involvement was defined as the presence of at least one of these manifestations. Some of these criteria (oxygen therapy, intravenous fluid resuscitation, and hospitalization) reflect management decisions rather than clinical signs alone and may depend on local practice. To explore how each type of systemic manifestation was distributed according to the number of stings, an exploratory two-graph receiver operating characteristic (TG-ROC) analysis was performed [11,12]. Unlike a conventional ROC curve, which plots sensitivity against 1 − specificity, TG-ROC plots sensitivity and specificity separately as functions of every possible cut-off value, so that the trade-off between them can be read directly on the sting-count scale. For this exploratory analysis, each domain was represented by a single binary indicator: digestive symptoms, neurological symptoms, oxygen therapy (respiratory domain), and intravenous fluid resuscitation (hemodynamic domain). For each indicator, patients with and without the indicator were compared, excluding patients with missing data. Analyses were performed with the pROC package in R. This representation was chosen because sting count is a continuous exposure without an established cut-off in this setting. For each type of manifestation, the cut-off maximizing the Youden index (sensitivity + specificity − 1) was reported [13]. These cut-offs were considered hypothesis-generating and were not intended as clinical decision thresholds.

## Results

### Population characteristics

A total of 7,236 visits related to bee stings were recorded at the Emergency Department (ED) of Cayenne Hospital (Fig 1). After reviewing medical records, 143 cases met the inclusion criteria and were retained for analysis. The mean annual incidence was 10.5 ± 2.8 cases per 100,000 inhabitants in the geographic study area (S1 Fig). The mean age of patients was 45 ± 20 years, with 10% under 15 years. Men accounted for 71% of the study population. A prior allergy to hymenopteran stings was reported in only 4 cases (2.8%).

**Fig 1:**
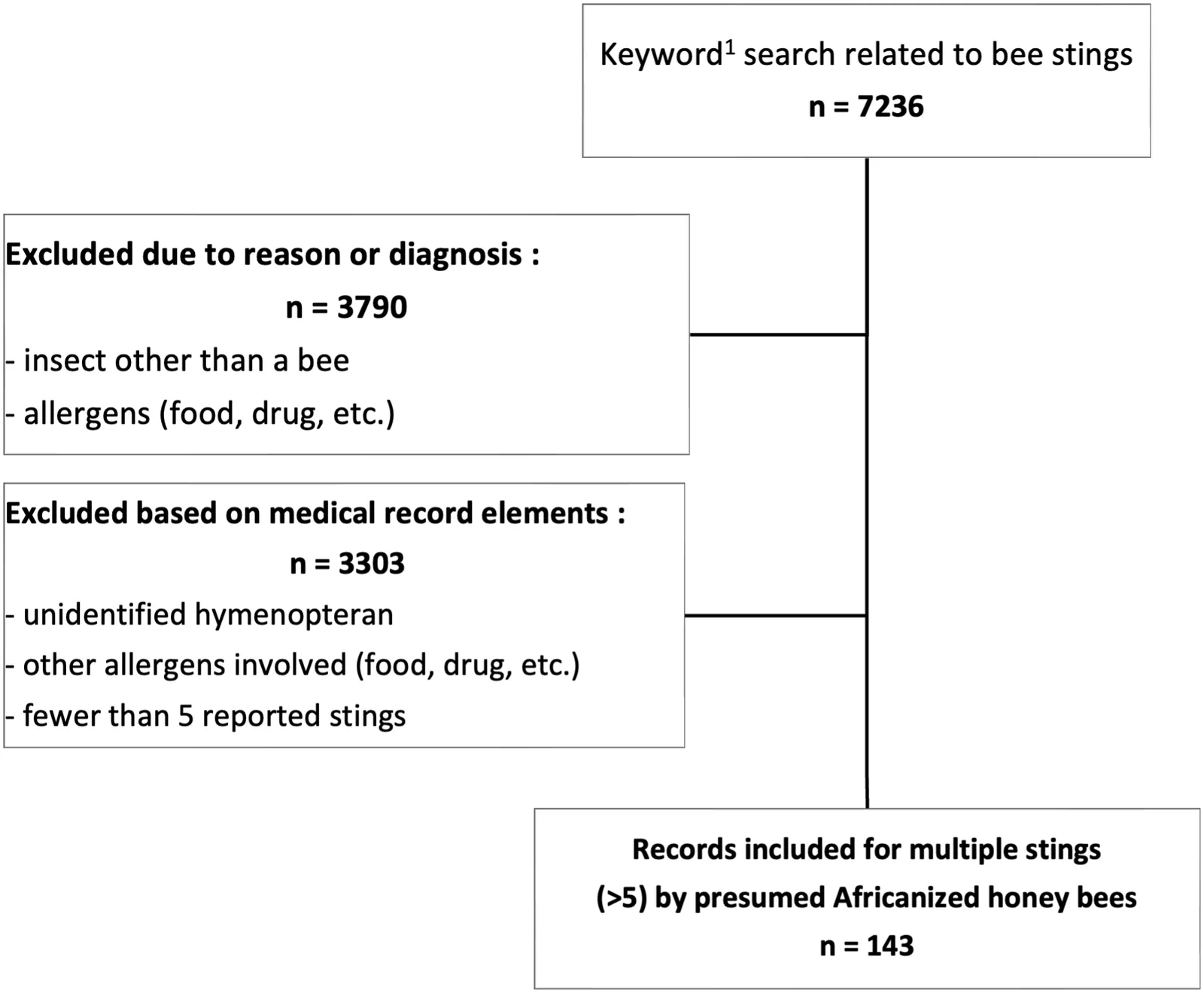
Study population flowchart. ^1^Keywords: “bee,” “insect,” “hymenoptera,” “allergy,” “anaphylaxis,” and “sting”.

### Characteristics of the stings

The majority of stings occurred during the dry season (July–December, 72%, Fig 2). All stings occurred during daytime; 23% were work-related accidents, and 41% involved multiple victims. Median number of stings was 50 [20–150] (S2 Fig). The head was the most commonly affected site (57%), followed by upper limbs (43%). Thirteen attacks (9.1%) were diffuse, affecting the entire body, with a mean of 407 ± 333 stings (Fig 2).

**Fig 2:**
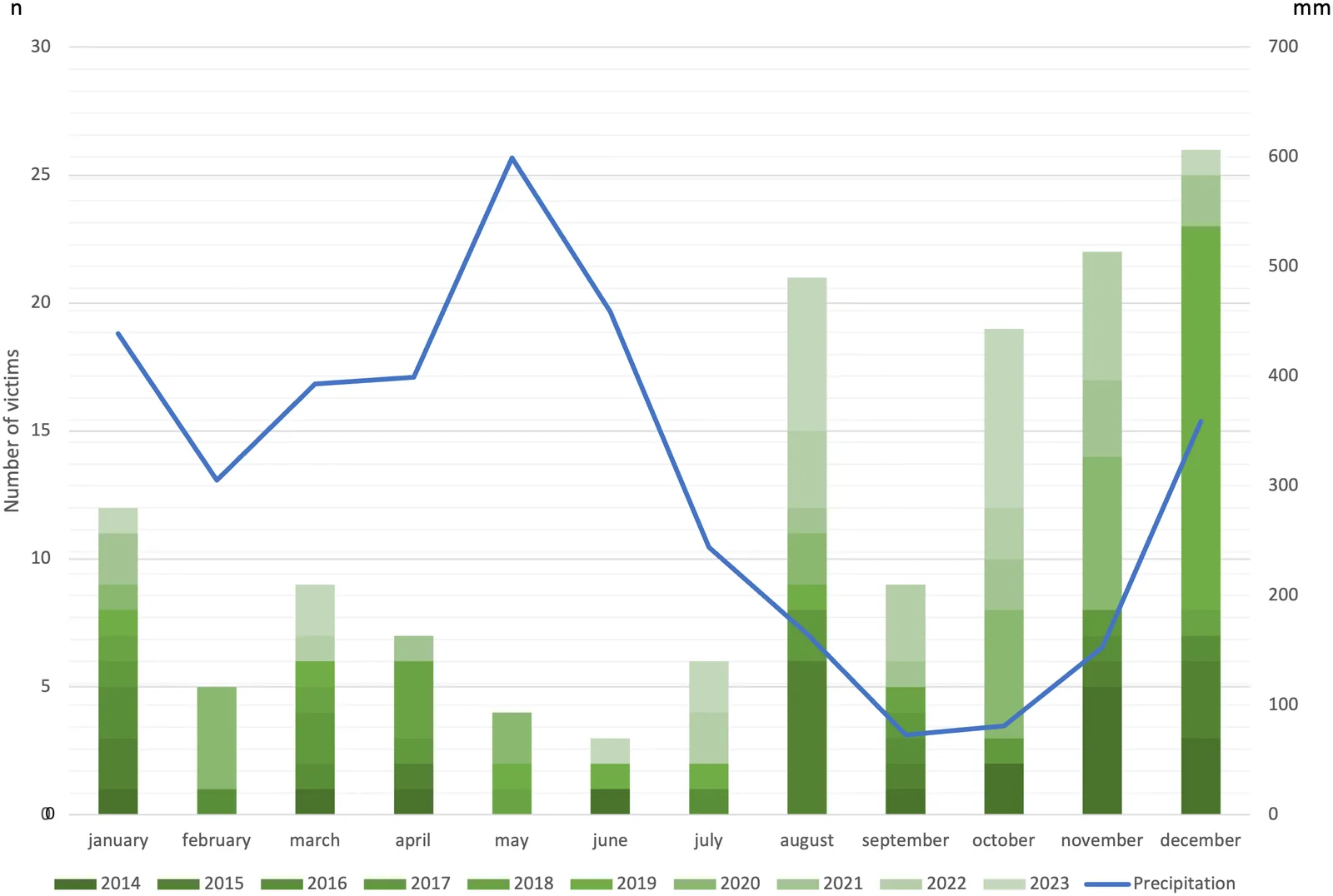
Monthly distribution of stings and monthly rainfall mean over ten years.

Clinical, biological and management characteristics of the study population is presented in the Table 1. Among the 143 patients included in the study, 40% (n = 57) presented systemic manifestations. The length of stay in the ED was 253 [150–475] minutes (S3 Fig). The main symptoms reported were erythema (n = 58, 41.4%), hives (n = 51, 36.4%), edema (n = 46, 32.9%) and digestive symptoms (n = 46, 32.9%). A prehospital medicalized team was deployed for 46 (32.4%) patients, fluid resuscitation and epinephrine was employed for respectively 33 (23.2%) and 41 (28.9%) patients. At the end of the emergency room management, 26 (18.4%) patients were hospitalized.

**Table 1:** Clinical, biological and management characteristics of the study population.

	Total n = 143
**Patients characteristics**	
Age	45.19 ± 20.48
Sex (Male)	101 (71.1%)
**Admission vitals signs**	
Glasgow score <15	4 (2.8%)
Heart rate 60–100	71 (50%)
Mean blood pressure <65mmHg	5 (3.5%)
Oxygen saturation <95%	20 (14.1%)
**Clinical symptoms**	
Number of stings	50 [20-150]
Widespread stings	13 (9.2%)
Pruritus	44 (31.4%)
Erythema	58 (41.4%)
Hives	51 (36.4%)
Edema	46 (32.9%)
Pharyngeal edema	5 (3.6%)
Digestive symptoms	46 (32.9%)
Neurological symptoms	12 (8.6%)
**Healthcare management**	
EMS	46 (32.4%)
Peripheral venous access	88 (62.4%)
Fluid resuscitation	33 (23.2%)
Oxygen support	17 (12%)
Analgesics	83 (58.5%)
Antihistamine	103 (72.5%)
Corticoids	92 (64.8%)
Epinephrine	41 (28.9%)
Hospitalisation	26 (18.4%)
**Biology examinations**	**N = 81 (56,6%)**
Leukocytes > 10 000/l	51 (63.8%)
Neutrophils >7 700/l	45 (56.2%)
Urea >8.1 mmol/l	6 (7.5%)
Creatinine >104 µmol/L	32 (40%)
AST > 41 U/l	16 (31.4%)
ALT > 40 U/l	12 (17.4%)
CK > 308 U/l	23 (43.4%)
CRP > 5 mg/l	10 (16.9%)
Troponin (Tn) > 0.014 ng/L	23 (57.5%)
Lactates > 2N	20 (51.3%)

EMS: Emergency Medical Service; AST: Aspartate aminotransferase; ALT: Alanine aminotransferase; CK: Creatine Kinase; CRP: C-reactive protein.

The specificities and the description of the pediatric (<15 years) population is presented in the Table 2. In this population, the median age was 6.0 [3.0-9.0] years old with a weight of 18.3 [14.4-34.6] kilograms. They presented 17.5 [10.0-23.7] stings, i.e., 0.66 [0.46-1.6] stings/kilogram. For the post-emergency room management, 4 (28.6%) were hospitalized including 3 (21.4%) in Intensive care unit. No hemodynamics failures were reported but the main symptoms recorded were digestive symptoms (n = 7, 50%) and erythema (n = 6, 42.9%).

**Table 2:** Clinical, biological and management characteristics of the pediatric population.

Variables	N = 14^1^
**Patients Characteristics**	
Age	6.0 [3.0-9.0]
Sexe (Male)	7.0 (50.0%)
Weight (kg)	18.3 [14.4 - 34.6]
Background of Allergy	0.0 (0.0%)
**Admission vitals signs**	
Glasgow score <15	1.0 (7.1%)
Heart Rate	122.7 (±16.8)
Mean blood pressure <65mmHg	0.0 (0.0%)
Oxygen saturation <95%	0.0 (0.0%)
**Clinical symptoms**	
Number of stings	17.5 [10.0 – 23.7]
Number of stings/kilograms	0.66 [0.46 – 1.6]
Widespread stings	1.0 (7.1%)
Pruritus	3.0 (21.4%)
Erythema	6.0 (42.9%)
Hives	5.0 (35.7%)
Edema	5.0 (35.7%)
Pharyngeal edema	0.0 (0.0%)
Digestive symptoms	7.0 (50.0%)
Neurological symptoms	2.0 (14.3%)
**Healthcare management**	
EMS	2.0 (14.3%)
Peripheral venous access	5.0 (35.7%)
Fluid resuscitation	2.0 (14.3%)
Epinephrine	4.0 (28.6%)
Oxygen support	0.0 (0.0%)
Analgesics	4.0 (28.6%)
Antihistamine	7.0 (50.0%)
Corticoids	8.0 (57.1%)
Biology examinations	8.0 (57.1%)
Hospitalisation	4.0 (28.6%)
Intensive Care Unit	3.0 (21.4%)
^1^n (%); Median [EI] (Mean ± ET)	

### Exploratory analysis: Number of stings and systemic involvement

As reported above, 57 patients (40%) presented at least one systemic manifestation. The exploratory TG-ROC analysis of sting count for each of the four indicators is shown in Fig 3. Solid lines represent sensitivity and dashed lines specificity across all sting-count cut-offs; vertical lines indicate the cut-offs maximizing the Youden index:

**Fig 3:**
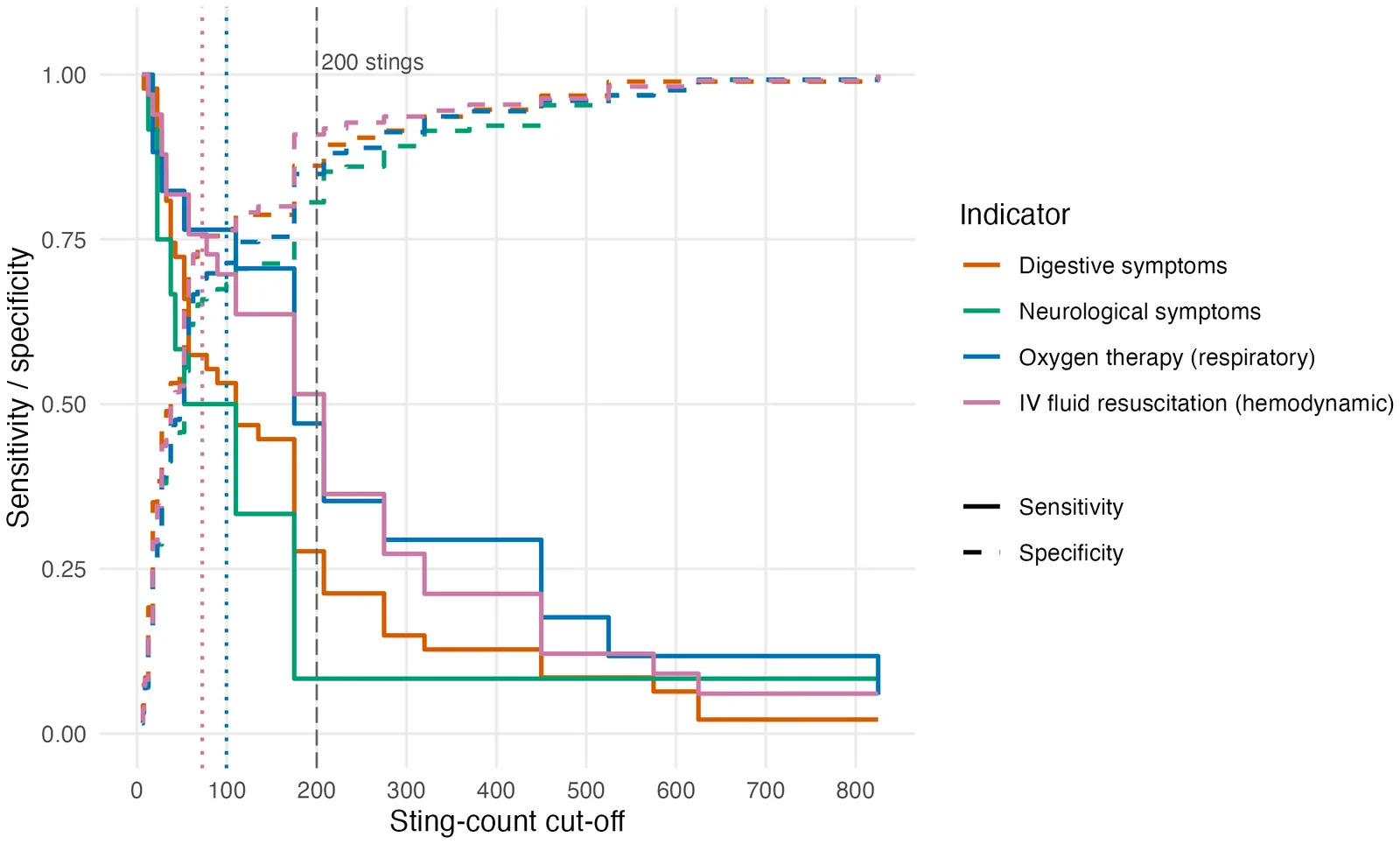
Two-graph ROC (TG-ROC) representation of the sensitivity (solid lines) and specificity (dashed lines) of sting-count cut-offs for four binary indicators of systemic involvement: digestive symptoms, neurological symptoms, oxygen therapy (respiratory) and intravenous fluid resuscitation (hemodynamic). For each indicator, patients with and without the indicator were compared among patients with available data. Vertical dotted lines indicate the exploratory cut-offs maximizing the Youden index (72 and 99 stings).

- Digestive symptoms and fluid resuscitation (hemodynamic): approximately 72 stings (respectively sensitivity/specificity 0.57/0.75 and 0.76/0.75).
- Neurological symptoms and oxygen therapy (respiratory): approximately 99 stings (respectively sensitivity/specificity 0.50/0.68 and 0.76/0.71).

At the conventional 200-sting threshold, sensitivity was below 0.40 for all four indicators, meaning that most patients presenting each indicator had received fewer than 200 stings. Conversely, specificity at the exploratory cut-offs did not exceed 0.76, so that many patients above these values did not present the corresponding indicator.

## Discussion

This study provides the first epidemiological description of massive bee attacks in French Guiana, with a detailed analysis of clinical outcomes and systemic manifestations. Over ten years, 143 cases were identified at Cayenne Hospital. Mass bee attacks have also been reported in Brazil [14,15], where they have been related to demographic growth and urban expansion into natural areas [1,16,17]. Seasonal variation was also observed, with more attacks during the dry season, consistent with increased flowering, bee activity, and swarming [15,18,19]. The overall incidence rate in our study (10.5/100,000/year) was lower than in Brazil [2,20], possibly reflecting stricter inclusion criteria (≥5 stings), lower apicultural density (0.2 vs 5 kg/m^2^ honey production), and our monocentric design. Work-related accidents accounted for 23% of cases, and the majority of victims were gardeners, tree trimmers, and construction workers, rather than professional beekeepers [21].

Mortality was low (0.7%, n = 1), similar to Brazilian reports (0.3%) [18]. While traditional thresholds define severe envenomation above 200 stings [3,6], our exploratory analysis showed that systemic manifestations were observed at lower sting counts (exploratory cut-offs of 72–99 stings). These exploratory cut-offs should not be interpreted as clinical decision thresholds, but they highlight the importance of early recognition of organ involvement. Due to retrospective analysis, the severity of envenomations regarding the patients weight was difficult to assess because of numerous missing data. Nevertheless, our analysis on pediatrics presenting no missing data on the weight, reported a low level of envenomation on our population regarding the literature [3]. Systemic manifestations were frequent (40%), exceeding the 1–4% anaphylaxis rates reported in the literature [3,22,23]. This discrepancy may be due to selection bias (≥5 stings), referral of severe cases, or an anaphylactoid reaction to high venom load [5,24,25]. Melittin-induced cytotoxicity, mast cell degranulation, and biogenic amine release at high doses likely contribute to renal, hepatic, and cardiac dysfunction. These findings underscore the need for early identification of at-risk patients and intensive monitoring, including serial laboratory assessments of renal, hepatic, and cardiac biomarkers. Some of these failures happen in a second time during the following of the most severe patients but were not analyzed in this present study [17].

The TG-ROC shows that sensitivity decreased steeply between approximately 70 and 200 stings for all four indicators, so that most patients with these indicators had received fewer than 200 stings. The overlap between the curves and the modest specificity at the exploratory cut-offs indicate that the number of stings alone discriminated poorly, which is expected given the contribution of allergic mechanisms, individual susceptibility (age, weight, comorbidities), and the imprecision of retrospective sting counts. Moreover, two of the four indicators (oxygen therapy and fluid resuscitation) are treatments, which reinforces the descriptive nature of this analysis. These observations are consistent with data on other Hymenoptera. in a Chinese cohort of 1,091 patients stung by wasps, in-hospital mortality was 5.2% among patients with more than 10 stings versus 1.0% among those with 10 stings or fewer, and elevated serum creatinine was also more frequent above 10 stings [26]. In another Chinese cohort of swarm wasp stings, a cut-off of more than 29 stings identified fatal cases with a sensitivity of 72.7% and a specificity of 89.4%, and the Chinese expert consensus used by these authors classifies envenomations with more than 30 stings as severe [27]. These values, much lower than those reported for honey bees, probably reflect differences in venom composition and in the amount of venom delivered per sting, as wasps, unlike honey bees, do not lose their stinger and can sting repeatedly. For honey bees, the classical thresholds of 200 stings for severe envenomation and 500 stings for potentially fatal envenomation in adults are derived mainly from estimates of venom lethality and from case series [3,6]. Taken together, these data suggest that sting-count thresholds are species-specific and are better viewed as a continuum of risk than as a dichotomous cut-off. To our knowledge, no previous study has described the sensitivity and specificity of sting count across its full range in honey bee envenomation, which limits direct comparison with our findings.

Prompt supportive care remains critical for prognosis [15,17]. Interventions should include stinger removal, aggressive hydration, hemodynamic support, and monitoring guided by severity. Novel antivenoms under development in Brazil are promising [19,28,29], and their evaluation in French Guiana could further improve outcomes. Limitations include the monocentric design and potential underestimation of envenomation burden in remote regions. Moreover, the retrospective nature of data collection may have led to incomplete clinical documentation or underreporting of mild cases. Another limitation is the retrospective estimation of the number of stings. Sting counts were extracted from routine medical records and were generally based on patient report and clinical assessment. As the estimation method was not standardized or systematically documented, some degree of measurement error or misclassification is possible, which may have influenced the analyses involving sting count. In addition, some of the operational criteria for systemic involvement (oxygen therapy, intravenous fluid resuscitation, and hospitalization for gastrointestinal involvement) are management decisions rather than clinical signs per se; they may depend on local practice and introduce a degree of circularity between management and outcome. Objective physiological criteria alone would have been preferable but were inconsistently recorded in this retrospective dataset, so the composite outcome should be interpreted as a descriptive marker of clinical severity rather than as a validated endpoint. The absence of entomological confirmation of bees species represents also an important limitation. Broader epidemiological surveillance, particularly in Kourou and Saint-Laurent-du-Maroni, combined with the upcoming toxicovigilance framework in French Guiana, should enable comprehensive reporting of massive bee stings [30].

## Conclusion

This study provides the first epidemiological description of massive bee presumed to be Africanized honey bees envenomations in French Guiana. Exploratory analyses suggested that systemic manifestations may occur at sting counts below the conventional 200-sting threshold. These findings should not be interpreted as a validated clinical cutoff and require confirmation in prospective multicenter studies.

## Supporting information

S1 Fig Number of attacks by year. (TIF)

S2 Fig Distribution of the number of stings in the study population. (TIF)

S3 Fig Patients length of stay in the emergency department. (TIF)
